# IL-10 mRNA Engineered MSCs Demonstrate Enhanced Anti-Inflammation in an Acute GvHD Model

**DOI:** 10.3390/cells10113101

**Published:** 2021-11-10

**Authors:** Cuiping Zhang, Mina Delawary, Peng Huang, Jennifer A. Korchak, Koji Suda, Abba C. Zubair

**Affiliations:** 1 Center for Regenerative Medicine and Department of Laboratory Medicine and Pathology, Mayo Clinic, Jacksonville, FL 32224, USA; Zhang.Cuiping@mayo.edu (C.Z.); Huang.Peng@mayo.edu (P.H.); Korchak.Jennifer@mayo.edu (J.A.K.); 2 Cell Therapy Research Laboratories, Daiichi Sankyo, Co., Ltd., Tokyo 1408710, Japan; delawary.mina.h7@daiichisankyo.co.jp (M.D.); suda.koji.gb@daiichisankyo.co.jp (K.S.)

**Keywords:** mRNA engineered MSCs, interleukin 10, immunosuppression, graft versus host disease

## Abstract

Mesenchymal stem cells (MSCs) are used in various studies to induce immunomodulatory effects in clinical conditions associated with immune dysregulation such as graft versus host disease (GvHD). However, most of these clinical trials failed to go beyond early phase 2 studies because of limited efficacy. Various methods have been assessed to increase the potency of MSCs. IL-10 is an anti-inflammatory cytokine that is known to modulate immune responses in GvHD. In this study, we evaluated the feasibility of transfecting IL-10 mRNA to enhance MSC therapeutic potential. IL-10 mRNA engineered MSCs (eMSCs-IL10) maintained high levels of IL-10 expression even after freezing and thawing. IL-10 mRNA transfection did not appear to alter MSC intrinsic characteristics. eMSCs-IL10 significantly suppressed T cell proliferation relative to naïve MSCs in vitro. In a mouse model for GvHD, eMSCs-IL10 induced a decrease in plasma level of potent pro-inflammatory cytokines and inhibited CD4+ and CD8+ T cell proliferation in the spleen. In summary, our studies demonstrate the feasibility of potentiating MSCs to enhance their immunomodulatory effects by IL-10 mRNA transfection. The use of non-viral transfection may generate a safe and potent MSC product for treatment of clinical conditions associated with immune dysregulation such as GvHD.

## 1. Introduction

Mesenchymal stem/stromal cells (MSCs) have been shown to have immune modulatory, angiogenic, and antiapoptotic effects by secreting exosomes, chemokines, cytokines, and growth factors that promote cell regeneration and survival [1,2,3]. Although MSCs are known to be well tolerated in vivo due to their low HLA antigen expression [4,5], some reports have shown that they may be subjected to immune clearance and first-pass effect when administered intravenously [6,7]. They have been widely used in many cell-based clinical trials. Over 10,000 clinical trials that involve a variety of human diseases such as heart disease, stroke, cancer, diabetes, and COVID-19 are on-going or have been completed [8].

Because MSCs can be easily acquired from different tissues and manipulated in vitro, in the last two decades, many studies have used MSCs as transgene carriers by genetically modifying them to enhance their therapeutic efficacy by overexpressing specific cytokines, such as VEGF [9] and IL-10 [10]. The genetic modification involves viral and non-viral mediated methods. Although viral transduction is more efficient and can lead to stable gene expression, its clinical utility is limited because it may cause chromosomal instability [11] which can result in malignant transformation. MSCs are mainly considered to function via a hit-and-run mechanism, with an in vivo life span ranging from days to a few weeks [7,12]. Despite this, the therapeutic effects of MSCs have been observed to last for a long time. Thus, mRNA transfection resulting in transient target protein overexpression is a potential way to engineer MSCs without altering their inherent mechanism of action. Transient target protein overexpression will ensure limited and controlled physiological effects of the engineered MSCs (eMSCs). Hence, we evaluated the feasibility and stability of mRNA eMSCs in vitro and their therapeutic potential in vivo.

MSCs’ immunomodulatory effects can be achieved by cell-to-cell contact and paracrine activity [13]. Numerous immunoregulatory factors released by MSCs participate in this process, including indoleamine 2,3-dioxygenase (IDO), prostaglandin E2 (PGE2), interleukin 10 (IL-10), and nitric oxide (NO) [14]. Among them, IL-10, which is mainly secreted by immune cells, is widely known as an anti-inflammatory cytokine which can limit T cell expansion and inhibit the production of pro-inflammatory cytokines [15].

Graft versus host disease (GvHD) is an adverse immunologic phenomenon observed after allogenic hematopoietic stem cell transplant [16]. Previous reports suggest administration of MSCs immediately after allogenic bone marrow transplant prevents the incidence of GvHD [17]. Similarly, MSCs have been used in clinical trials to treat GvHD. However, the therapeutic effectiveness reported in most of these studies is limited [18]. IL-10 has been shown to have beneficial effects in the treatment of immune and inflammatory disorders, such as Crohn’s disease [19] and psoriatic arthritis [20]. Although IL-10 is one of the important immunomodulatory cytokines in MSC paracrine activities, it is not well-expressed by bone marrow derived MSCs (BMSCs). To enhance the therapeutic efficacy of MSCs for immune-mediated diseases, we engineered MSCs to overexpress human IL-10 (hIL-10) and evaluated their therapeutic efficacy in an acute GvHD (aGvHD) model.

The therapeutic potential of DNA engineered MSCs overexpressing IL10 has been studied in the aGvHD [21], arthritis [22], lung ischemia-reperfusion injury [23], traumatic brain injury [24], and middle cerebral artery occlusion [25] in in vivo models. Unlike DNA engineered MSCs, the application of mRNA engineered MSCs overexpressing IL-10 (eMSCs-IL10) has not been reported in a GvHD model.

**Figure 1:**
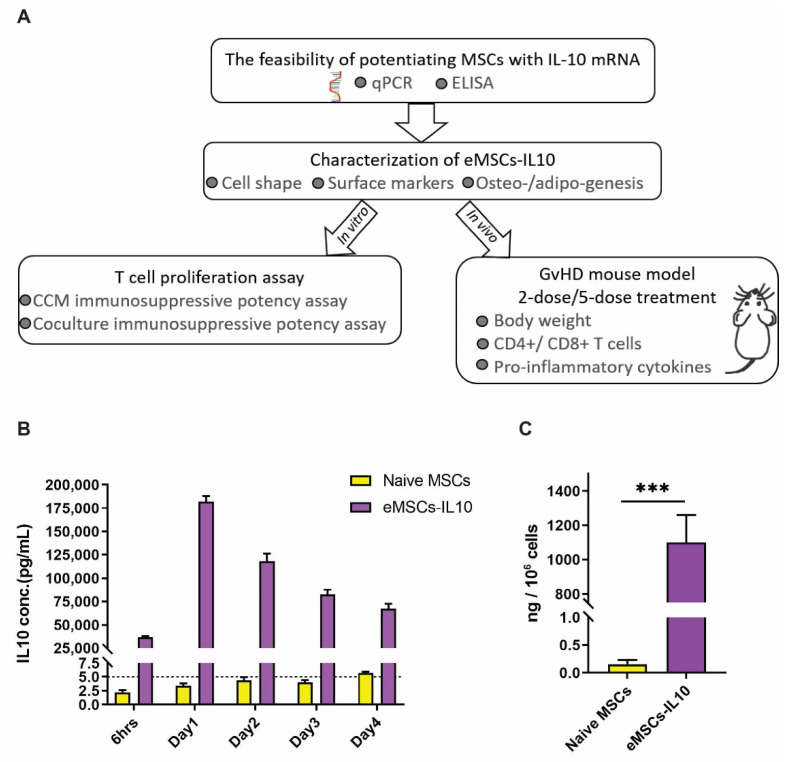
Characteristics of IL-10 secretion from eMSCs -IL10. (**A**) Summary schematic diagram illustrating the workflow of evaluating the immunosuppressive effects of eMSCs-IL10. (**B**) IL-10 secretion from eMSCs-IL10 lasts for at least 4 days. The culture supernatant was collected after 6 h and days 1, 2, 3, and 4 after mRNA transfection. A robust release of IL-10 after mRNA transfection was observed and the peak expression appeared after 1-day culture. Data shown as mean ± SD (*n* = 4). (**C**) The amount of IL-10 released from thawed eMSCs-IL10 after 1-day culture. The cryopreserved naïve MSCs and eMSCs-IL10 were thawed and then cultured for 24 h. Data presented as mean ± SD (*n* = 3), *** *p* < 0.001.

Here, we generated eMSCs-IL10 and evaluated their immunosuppressive effects in vitro and in vivo (Figure 1A). First, we conducted a time course experiment to detect the IL-10 expression over time and confirmed the stability of IL-10 secretion even after cryopreservation. In vitro, T cell proliferation assays were used to demonstrate the immunosuppressive function of eMSCs-IL10. Our data showed that eMSCs-IL10 significantly inhibited CD4+ and CD8+ T cell proliferation and reduced some pro-inflammatory cytokine levels in an aGvHD model. Collectively, eMSCs-IL10 exhibit robust IL-10 secretion, exert immunosuppressive effects both in vitro and in vivo, and may provide a more effective therapy when used in the clinical management of GvHD without concerns of MSC maltransformation.

## 2. Materials and Methods

### 2.1. Animals

All experimental procedures were approved by Daiichi Sankyo’s Institutional Animal Care and Use Committee (Approval number: 2000516) and UNITECH Co. (Chiba, Japan)’s Institutional Animal Care and Use Committee (Approval number: AGR DIS-200526A-30). The study was carried out in accordance with the Animal Experimentation Guidelines of UNITECH Co., Ltd. which is based on Japanese laws and guidelines, including the Act on Welfare and Management of Animals and the fundamental guidelines issued by the Ministry of Health, Labor, and Welfare and related activities. The study was also carried out in compliance with the ARRIVE guidelines.

Female CB17/Icr-Prkdc^scid^/CrlCrlj mice at 5 weeks old were purchased from Charles River Laboratories Japan, Inc. (Kanagawa, Japan). Female C57BL/6J mice at 7 weeks old were purchased from CLEA Japan, Inc. (Tokyo, Japan). The animals were used for this study after an acclimation period of 5 days. CB17/Icr-Prkdc^scid^/CrlCrlj mice were housed individually, and C57BL/6J mice were housed at four per cage, with food and water made available ad libitum. The mice were maintained under pathogen-free conditions with controlled humidity (40–65%), temperature (22–26 °C), and 12 h light/dark cycles.

### 2.2. Custom mRNA Synthesis

CleanCap^®^ EGFP mRNA (Catalog # L-7601) was purchased from TriLink BioTechnologies (San Diego, CA, USA). The open reading frame (ORF) sequence for hIL-10 was acquired from NCBI (Homo sapiens interleukin 10, mRNA (NM_000572.3)), and mature IL-10 mRNA was synthesized by TriLink BioTechnologies, including T7 promoter, 5′ UTRs, 3′ UTRs, and poly-A tail.

### 2.3. MSC Culture and Time Course Experiment

Human MSCs were generated from commercial de-identified bone marrow (Catalog# ABM002) purchased from AllCells (Alameda, CA, USA). The donor was a healthy 28-year-old Caucasian male. MSCs were initially isolated and expanded to passage 1 (P1) in the complete growth medium composed of MEM α (Gibco, Grand Island, NY, USA, catalog# 12561072) supplemented with 5% human platelet lysate (hPL) (Sexton Biotechnologies, Indianapolis, IN, USA) and 1% GlutaMAX™ (Gibco, catalog# 35050061) using an automated cell expansion system (Quantum, Terumo BCT, Lakewood, CO, USA) as previously described [26]. The P1 MSCs were cryopreserved at 10 × 10^6^ cells/mL and then expanded and cultured in complete growth medium using cell culture flasks and plates in a 5% CO_2_, 37 °C incubator. The MSCs used in our experiments were within passage 4.

BMSCs at passage 3 were seeded into 6-well plates. Transfection was conducted with EGFP mRNA or IL-10 mRNA using Lipofectamine™ MessengerMAX™ Reagent (Thermo Fisher Scientific, Waltham, MA, USA, catalog# LMRNA015) and Opti-MEM™ I Reduced Serum Medium (Gibco, catalog# 31985070) when the cell confluence reached about 80%, as per the manufacturer’s protocols. EGFP mRNA or hIL-10 mRNA at a concentration of 1.25 µg/mL and 1.875 µL/mL of Lipofectamine MessengerMAX Reagent were added per well. After 1, 2, 4, and 6 days of EGFP mRNA transfection, the images were taken. After 6 h, 1, 2, 3, and 4 days of IL-10 mRNA transfection, the conditioned medium was collected for ELISA assay. Similarly, naïve MSCs were treated the same way except that they were not subjected to mRNA transfection.

### 2.4. Preparation of Conditioned Culture Medium (CCM)

MSCs were cultured and transfected as described above. After 4 h of transfection, cells were cryopreserved in CryoStor^®^ CS10 (StemCell Technologies, Vancouver, BC, Canada, catalog# 07930) according to the manufacturer’s directions. The cryopreserved cells at passage 4 were used for preparation of CCM. Two hundred thousand thawed eMSCs-IL10 or naïve MSCs were seeded per well in a 6-well plate, each well containing 2 mL of complete growth medium. The CCM was collected after 24 h of MSC culture, then centrifuged at 1500 rpm for 10 min at 4 °C to remove cell debris, aliquoted, and stored at −80 °C. The CCM samples were used for ELISA and the in-vitro T cell proliferation assays.

### 2.5. MSC Phenotyping

The frozen naïve MSCs and eMSCs-IL10 were thawed for this assay. An MSC Phenotyping Kit (Miltenyi Biotec, Bergisch Gladbach, Germany, catalog# 130-125-285) was used to detect MSC surface molecule expression according to the manufacturer’s protocols. The antibody cocktail consists of CD73, CD90, CD105, CD45, CD34, CD19, CD14, and HLA-DR. A MACSQuant Analyzer 16 flow cytometer was used to collect the data. A MACS^®^ Comp Bead Kit (Miltenyi Biotec, Bergisch Gladbach, Germany, catalog# 130-104-187) was used for compensation before each run.

### 2.6. Differentiation of MSCs

Naïve MSCs and eMSCs-IL10 at passage 4 were seeded into 24-well plates at a density of 8000 cells per well. For adipogenic differentiation, when the cells reached 90~100% confluence, the complete adipogenesis differentiation medium (Gibco, Grand Island, NY, USA, catalog# A10070-01) was added and changed every 3–4 days. After 11 days, the cells were stained with oil red O. Briefly, cells were fixed with 4% formaldehyde solution (Fisher scientific, Fair Lawn, NJ, USA, catalog# SF98-4) for 1 h at room temperature, and stained with freshly prepared oil red O working solution containing 3 parts of 0.5% oil red O solution (Sigma, St Louis, MO, USA, catalog# O1391) and 2 parts of DPBS (Gibco, catalog# 14190-250) for 20 min. For osteogenic differentiation, the cells at roughly 50% confluency were fed with complete osteogenesis differentiation medium (Gibco, Grand Island, NY, USA, catalog# A1007201) every 3–4 days. After 19 days of inducing differentiation, the alizarin red S staining was processed. Briefly, after fixing cells with 4% formaldehyde solution for 1 h, 1% alizarin red S solution (Sigma, St Louis, MO, catalog# A5533) was used for staining. The corresponding controls were cultured in the complete growth medium and processed with the same staining procedure. Images were captured with bright field microscopy.

### 2.7. Quantitative Real-Time PCR

Total RNA was isolated from naïve MSCs or eMSCs-IL10 using the RNeasy Plus Mini Kit (Qiagen, Hilden, Germany, catalog# 74134). The concentration of RNA was determined with a NanoDrop 2000 Spectrophotometer (Thermo Fisher Scientific, Waltham, MA, USA). Reverse transcription was conducted with 70.2 ng of RNA using a QuantiNova Reverse Transcription Kit (Qiagen, catalog# 205411). The synthesized cDNA was diluted by 1:7 and used for real-time PCR. TaqMan Fast Advanced Master Mix (Applied Biosystems, Waltham, MA, USA, catalog# 4444963) and the following Gene Expression TaqMan Assays were used: IL-10 (Hs00961622_m1) and GAPDH (Hs02758991_g1). GAPDH was used as an internal control, and the expression of the targeted gene was determined by the 2^−ΔΔCt^ method. Each sample was evaluated in triplicate.

### 2.8. Enzyme-Linked Immunosorbent Assay (ELISA)

The conditioned medium from naïve MSCs or eMSCs-IL10 was collected at different time points, centrifuged at 1500 rpm at 4 °C for 10 min, aliquoted, and immediately stored at −80 °C. The medium was thawed on ice and then the concentration of secreted IL-10 was determined using the IL10 Human ELISA Kit (Aviva Systems Biology, San Diego, CA, USA, catalog# OKBB00193) according to the manufacturer’s instructions. The absorbance at 450 nm was read by a microplate reader (SpectraMax^®^ M2, Molecular Devices, Sunnyvale, CA, USA). Each sample was tested in duplicate.

### 2.9. CCM Immunosuppressive Potency Assay

Peripheral blood mononuclear cells (PBMCs) (Cellero, Wilmington, MA, USA, catalog#1001, Lot# 4914OC20) were seeded into a poly-L-ornithine coated 96-well plate at 40,000 cells per well and cultured in a 1:1 mixture of RPMI1640 medium (Gibco, catalog# 11875093) containing 10% FBS (Atlanta Biologicals, Flowery Branch, GA, USA, catalog# S11550) and 1% GlutaMAX™ and MEM α medium supplemented with 5% hPL and 1% GlutaMAX™. Phytohemagglutinin (PHA-P, PHA) (Sigma, catalog# L1668-5MG) at 10 µg/mL, rapamycin (InvivoGen, San Diego, CA, USA, catalog# tlrl-rap) at 10 µg/mL, and CCM from naïve MSCs or eMSCs-IL10 were added to the respective groups. For the groups with the IL-10 receptor α antibody (IL10 Ra AB) (R&D Systems, Minneapolis, MN, USA, catalog# MAB274), PBMCs were incubated with 80 µg/mL of IL10 Ra AB for 1.5 h prior to adding other treatments, PHA, and CCM-IL10 (100 ng/mL). After 3-day incubation, the cell viability was determined using the CellTiter-Glo^®^ Luminescent Cell Viability Assay Kit (Promega, Madison, WI, USA, catalog# G7571), which is based on ATP quantification. The data were normalized to the PBMC group stimulated with PHA. One-way ANOVA was used for statistical analysis (*n* = 4).

### 2.10. Coculture Immunosuppressive Potency Assay

This assay was performed using the protocols as previously described with minor modifications [27]. Briefly, MSCs were seeded into a 96-well plate with 10,000 cells/well for 1:5 cell ratio groups and 5000 cells/well for 1:10 cell ratio groups. After 2 h of incubation in the cell culture incubator, PBMCs (Cellero, Wilmington, MA, USA, catalog# 1001, lot# 4498NV19) were seeded into the wells containing MSCs at a density of 50,000 cells/well. PHA at 10 µg/mL, and rapamycin at 10 µg/mL were added to the respective groups. The 96-well plate was incubated in the cell culture incubator for 3 days, and then cell viability was measured using the CellTiter-Glo^®^ Luminescent Cell Viability Assay Kit. Each group was assessed in quintuplicate, and one-way ANOVA was used for statistical analysis.

### 2.11. The GvHD Model

Female CB17/Icr-Prkdc^scid^/CrlCrlj mice were used as recipients and female C57BL/6J mice were used as donors. Due to the major histocompatibility (MHC) class I mismatch of CB17/Icr-Prkdc^scid^/CrlCrlj mice (H2-d) and C57BL/6J mice(H2-b), injecting of the spleen single cell suspensions (splenocytes) from C57BL/6J mice into CB17/Icr-Prkdc^scid^/CrlCrlj mice was used to induce aGvHD and the acute graft-versus-host reaction was validated. The mice underwent a 5-day acclimation period before the start of the experiments. At Day 0, splenocytes were prepared by disrupting the spleens from four female C57BL/6J mice. Forty female CB17/Icr-Prkdc^scid^/CrlCrlj mice were allocated to five groups by randomized block design (*n* = 8 mice /group) to equalize body weight variance among groups. The groups were divided as follows: vehicle group, naïve MSCs 2-dose group, naïve MSCs 5-dose group, eMSCs-IL10 2-dose group, and eMSCs-IL10 5-dose group. Recipient mice were then injected with 5 × 10^6^ splenocytes/200 µL PBS via tail vein to induce aGVHD. The body weight of the recipient mice was measured daily from Day 0 to Day 16. Blood samples were collected on Day 7 and Day 16 for mice serum cytokine profiling and measurement of hIL-10 concentration. On Day 16, the mice were euthanized by exsanguination via the abdominal aorta and post vena cava under deep isoflurane anesthesia, and the spleens were collected for immunophenotyping.

### 2.12. MSC Treatment

To evaluate the immunosuppressive effects of MSCs, naïve MSCs or eMSCs-IL10 were administered via tail vein to recipient mice on Day 3 and Day 6 for the 2-dose treatment regimen, or on Day 3, 6, 9, 12, and 15 for the 5-dose treatment regimen. MSCs cryopreserved 4 h post-transfection were used for the preparation of MSCs suspension. Each suspension was adjusted to a concentration of 5 × 10^5^ cells/200 µL saline/mouse. In order to align the experimental conditions of each group, the vehicle group received 200 µL of saline for 5-dose, and the 2-dose MSCs-treated groups received 200 µL of saline on Day 9, 12, and 15.

### 2.13. FACS Analysis of Splenocytes in the GvHD Model

On Day 16, the mice were euthanized and the spleens were collected for immunophenotyping. Splenocytes were first incubated with an Fc-receptor blocking monoclonal antibody (BD Pharmingen, San Jose, CA, USA, catalog# 553142) at 4 °C for 5 min, then directly stained with H-2Kb-FITC (Invitrogen, Waltham, MA, USA, catalog# 11-5958-82), CD-4-PE (BD Pharmigen, catalog# 553049), and CD-8-BV421 (BD Biosciences, San Jose, CA, USA, catalog# 563898) at 4 °C for 15 min. Finally, the cells were washed with FACS buffer and analyzed with a MACSQuant Analyzer (Miltenyi Biotec, Bergisch Gladbach, Germany) using FlowJo software (BD Biosciences, San Jose, CA, USA, Version 10). All the CD4+ cells and CD8+ cells were also stained with H-2Kb, showing that these cells were derived from C57BL/6J mice (data not shown).

### 2.14. Human IL-10 Concentration in Mice Serum in the GvHD Model

To assess the secretion ability of eMSCs-IL10 in vivo, hIL-10 concentration was measured using the serum collected on the day following MSCs administration (Day 7 and Day 16). Human IL-10 was measured using the hIL-10 ProQuantum Immunoassay Kit (Thermo Fisher Scientific, Waltham, MA, USA, catalog# A35590), in accordance with the manufacturer’s standard protocol.

### 2.15. Serum Cytokine Profiling in the GvHD Model

The serum samples were collected on Day 7 and Day 16, and cytokines were measured by a Milliplex MAP Mouse Cytokine/Chemokine Magnetic Bead Panel (22 plex: Eotaxin, GM-CSF, IFNγ, IL-1α, IL-1β, IL-2, IL-4, IL-5, IL-6, IL10, IL-12 p40, IL12- p70, LIF, IL-13, IL-15, IL-17, MCP-1, MIP-1α, MIP-1β, MIP-2, MIG, TNFα) (Millipore, Darmstadt, Germany) and a Milliplex MAP Mouse TH17 Magnetic Bead Panel (5 plex: IL-17E/IL-25, IL-21, IL-22, IL-17A, IL-17F) (Millipore, Darmstadt, Germany), following the standard protocol provided by the vendor.

### 2.16. Statistical Analysis

All data were presented as mean ± SD. One-way ANOVA, two-way ANOVA, and two-tailed unpaired *t* tests were used for statistical analyses. The ELISA result analysis (Figure 1C) was conducted using two-tailed unpaired *t* tests. One-way ANOVA analyses with Dunnett’s multiple comparisons test were used in the in-vitro T cell proliferation assays (Figures 3 and 4). Two-way ANOVA with Dunnett’s multiple comparisons test was used in the analysis of mice body weight (Figure 5B). CD4+ and CD8+ T cell proliferation analyses (Figure 5C,D) and the analyses of mouse serum cytokine profiling (Figure 6A,B) were conducted using two-tailed unpaired *t* tests. All the calculations were conducted with GraphPad Prism (Version 9.0.1). *p* values less than 0.05 (* or #), *p* < 0.01 (**), *p* < 0.001 (*** or ###), and *p* < 0.0001 (**** or ####) were considered statistically significant.

## 3. Results

### 3.1. EGFP mRNA Engineered MSCs Are Viable and Show Stable GFP Expression

We began our studies with a feasibility experiment of transfecting EGFP mRNA transcripts into MSCs. After optimization, a final concentration in culture medium of 1.25 µg mRNA/mL and 1.875 µL Lipofectamine MessengerMAX/mL was found to limit the cytotoxic effects of the lipofectamine and mRNA transcripts while maintaining optimal overexpression of the target gene. As shown in Supplementary Materials Figure S1, a majority of the transfected MSCs showed visual GFP positivity. This indicates that it is feasible to transfect large sized mature mRNA fragments into MSCs with continuous protein expression of up to 6 days or possibly longer.

### 3.2. MSCs Transfected with IL-10 mRNA Express and Secrete IL-10 in High Concentration

Next, we transfected MSCs with hIL-10 and performed a time course experiment to determine IL-10 mRNA levels in IL-10 mRNA engineered MSCs (eMSC-IL10) and IL-10 protein secretion in the conditioned culture medium (CCM). First, we detected high levels of IL-10 mRNA in eMSCs-IL10 (Ct ≈ 17) while IL-10 mRNA was barely detectable in naïve MSCs (Ct > 37) by real-time PCR (Figure S2). As shown in Figure 1B, naïve MSCs barely secreted IL-10 (<5 pg/mL), whereas IL-10 concentration in CCM from eMSCs-IL10 reached up to approximately 40,000 pg/mL at 6 h post transfection. After one day of eMSCs-IL10 culture, the amount of IL-10 reached the peak and then started to decline. By day 4, the concentration dropped by more than 50% from the peak. However, this level is still roughly 13,200 times higher than that of naïve MSCs. Thus, hIL-10 was effectively secreted from eMSCs-IL10. Further studies will be needed to determine at what period the overexpressed IL-10 protein can no longer be detected.

We envisioned that our eMSCs-IL10 product would likely be cryopreserved before clinical application. We therefore optimized our manufacturing protocol in order to preserve optimal IL-10 secretion post cryopreservation and thawing. We detected robust secretion of IL-10 after 1-day culture of thawed eMSCs-IL10 (Figure 1C).

### 3.3. Characterization of eMSCs-IL10

**Figure 2:**
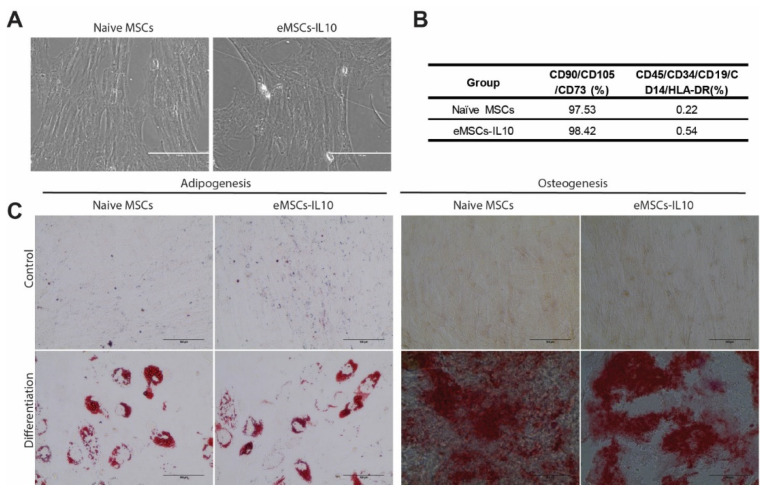
Characterization of naïve MSCs and eMSCs-IL10. (**A**) Representative images of naïve MSCs and eMSCs-IL10. Both naïve MSCs and eMSCs-IL10 displayed spindle or fibroblast-like morphology (scale bar: 200 µm). (**B**) Summary table showing MSC surface markers. Both naïve MSCs and eMSCs-IL10 were positive for cell surface markers CD90, CD105, and CD73 (>95%) and negative for molecules CD45, CD34, CD19, CD14, and HLA-DR (<5%). (**C**) Representative images of adipogenic and osteogenic differentiation of MSCs. Adipogenesis was demonstrated by oil red-O stain with distinct lipid vacuoles. Osteogenesis was exhibited by alizarin red S staining with mineral deposition (scale bar: 100 µm).

To characterize eMSCs-IL10, we evaluated their MSC identity using morphological analysis, flow cytometry, and adipogenic and osteogenic differentiation assays. Following the phenotypic guidelines established by the International Society for Cell & Gene Therapy (ISCT) [28], eMSCs-IL10 preserved their spindle or fibroblast-like adherent cell characteristics as compared with naïve MSCs (Figure 2A). MSCs were evaluated for the expression of CD105, CD73, and CD90 and the absence of CD45, CD34, CD19, CD14, and HLA-DR. We found no difference in the expression of MSC-related markers between eMSCs-IL10 and naïve MSCs (Figure 2B and Figure S3). In addition, both naïve MSCs and eMSCs-IL10 retained their ability to differentiate into adipogenic and osteogenic lineages (Figure 2C).

### 3.4. Engineered MSCs-IL10 Show Significant Immunosuppressive Properties In Vitro

**Figure 3:**
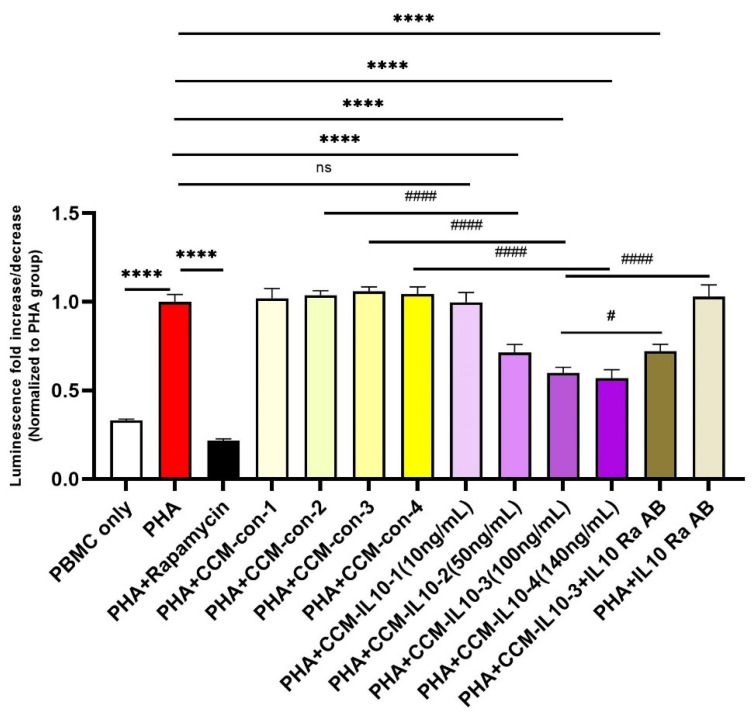
Immunosuppressive properties of CCM-IL10. Different concentrations of IL-10 in CCM-IL10 impact the immunosuppressive effects. PHA was used to activate T cells and induce proliferation, which can be mostly blocked by rapamycin. The immunosuppressive effects were observed using CCM-IL10 with IL-10 concentration ranging from 10 ng/mL to 140 ng/mL, while respective volumes of CCM-con did not cause significant decreases in cell viability. IL-10 receptor α antibody partly blocked the cell viability decrease induced by CCM-IL10 (100 ng/mL). Statistical differences were evaluated by one-way ANOVA. Data presented as mean ± SD (*n* = 4), * indicates groups compared with PHA only group, and # indicates comparisons with CCM-IL10 groups. **** *p* < 0.0001, # *p* < 0.05, and ##### *p* < 0.001.

Next, we evaluated the capacity of eMSCs-IL10 to suppress T cell proliferation by using PHA stimulated T cell proliferation assays. As shown in Figure 3, four different concentrations of overexpressed IL-10 from eMSCs-IL10 cultures (CCM-IL10) were evaluated in the CCM immunosuppressive potency assay (10 ng/mL, 50 ng/mL, 100 ng/mL, and 140 ng/mL) with the equivalent volumes of CCM from naïve MSCs (CCM-control, CCM-con) as controls. Among the groups, 10 ng/mL of CCM-IL10 did not cause a significant decrease of cell viability compared to the PHA only group. However, the other CCM-IL10 groups (50 ng/mL, 100 ng/mL, and 140 ng/mL) showed significant immunosuppressive effects when compared to either the PHA only group or corresponding CCM-con groups. In contrast, all four of the CCM-con groups appeared to have no significant immunosuppressive impact. The same results were observed using cluster formation assay (Figure S4, Videos S1 and S2).

**Figure 4:**
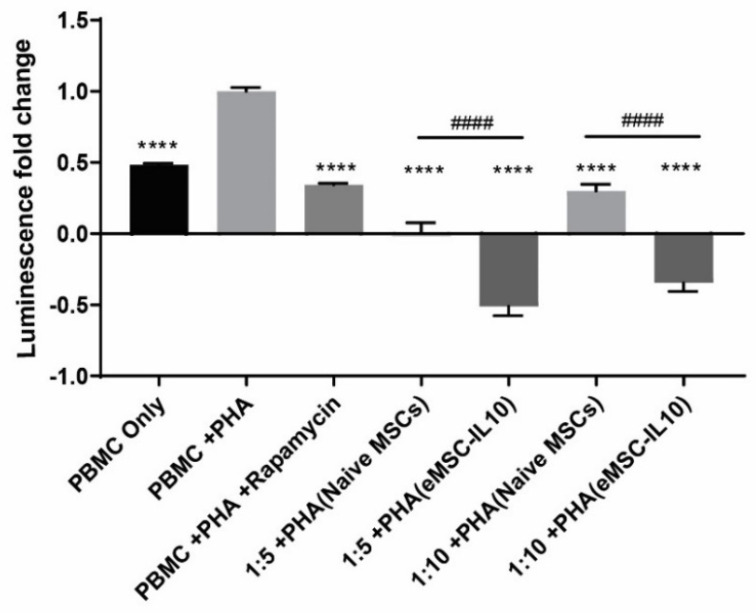
MSC immunosuppressive capacity in coculture potency assay. MSCs were incubated with PHA activated PBMCs in a 96-well plate with 1:5 and 1:10 cell ratios of MSC to PBMC. After 3 days of coculturing, the ATP content in each well was quantified and indicated by relative luminescence unit (RLU) value. The luminescence fold change was normalized to the PHA-stimulated PBMC group. Statistical differences were evaluated by one-way ANOVA. Data presented as mean ± SD (*n* = 5), * indicates groups compared with PHA stimulated PBMC group, and # indicates comparisons between naïve MSC coculture groups and eMSC-IL10 coculture groups (1:5 or 1:10). **** *p* < 0.0001 and #### *p* < 0.0001.

Additionally, we also assessed eMSCs-IL10’s immunosuppressive capacity in comparison with naïve MSCs via co-culturing the MSCs with PHA-activated PBMCs. As shown in Figure 4, in comparison to the PHA-only stimulated PBMC group, both naïve MSCs and eMSCs-IL10 coculture groups showed evident immunoinhibitory effects. Furthermore, regardless of the MSC to PBMC ratio, eMSC-IL10 exhibited greater immunosuppression compared to naïve MSCs (*p* < 0.0001 for both 1:5 and 1:10 ratios).

To test if the immunosuppressive effects produced by CCM-IL10 are specifically caused by IL-10, we blocked the IL-10 receptor on the PBMCs prior to the addition of CCM-IL10. As shown in Figure 3, pre-neutralization with IL-10 receptor antibody partly reversed the immunosuppressive effects of CCM-IL10 (100 ng/mL). This indicates that some other factors, in addition to IL-10, might have contributed to the CCM-IL10 induced immunosuppressive effects. Collectively, these results demonstrate that the secretion of IL-10 by eMSCs-IL10 effectively enhances the immunosuppressive function of MSCs in vitro.

### 3.5. eMSCs-IL10 Induce Significant Decreases in T Cell Expansion and Potent Pro-Inflammatory Cytokines in an aGvHD Mouse Model

**Figure 5:**
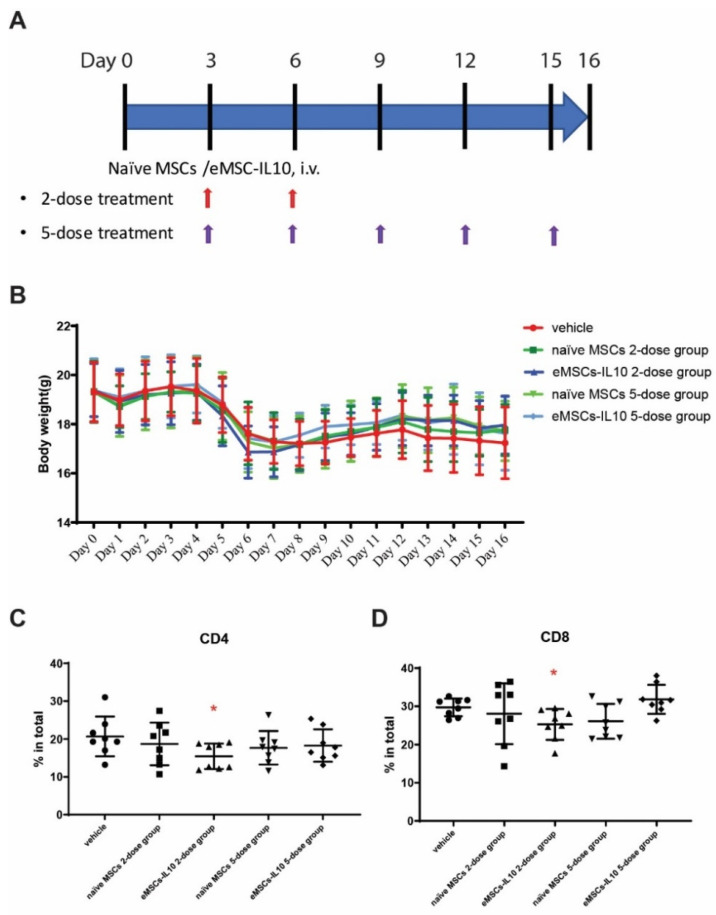
Engineered MSCs-IL10 significantly inhibited CD4+ and CD8+ T cells proliferation in the aGvHD mouse model. (**A**) Flow diagram of naïve MSCs and eMSCs-IL10 administration. In this experiment, two regimens were applied. For the two-dose treatment, MSCs were administered on Day 3 and Day 6. For the five-dose treatment, MSCs were administered every three days from Day 3 to Day 15 (total five doses). (**B**) Body weight of GvHD mice. There were no significant differences in body weight change among the groups. All the mice started to drop in body weight by Day 5, confirming the successful induction of GvHD. (**C**,**D**) Donor T cell infiltration in the host spleen. Donor CD4+ T cells (**C**) and donor CD8+ T cells (**D**) were significantly reduced in eMSCs-IL10 2-dose group on Day 17. Data expressed as mean ± SD, * *p* < 0.05.

Studies have shown that hIL-10 can act on various species such as mice despite the fact that mouse IL-10 is not active on human cells [29,30]. To evaluate the immunomodulatory effects of eMSCs-IL10 in vivo, we used an aGvHD mouse model. CB17/Icr-Prkdc^scid^/CrlCrlj mice were intravenously injected with splenocytes prepared from C57BL/6J mice to induce aGvHD (5 × 10^6^ cells per mouse, Day 0). Two-dose and five-dose treatment regimens were set up. For the 2-dose regimen, MSCs were administered on Day 3 and Day 6. For the 5-dose regimen, MSCs were administered every three days from Day 3 to Day 15, for a total of five times (Figure 5A). The capacity of eMSCs-IL10 to secrete hIL-10 in vivo was assessed. Twenty-four hours after the administration of the last dose, five of eight animals from the eMSCs-IL10 2-dose group and all mice from the eMSCs-IL10 5-dose group were evaluated for hIL-10 expression. Human IL-10 was detected at high levels in serum from mice in the eMSCs-IL10 2-dose group (148 ± 89 pg/mL) on Day 7 and the eMSCs-IL10 5-dose group (97 ± 15 pg/mL) on Day 16. Hence, we confirmed that eMSCs-IL10 can secrete a significant amount of hIL-10 in our GvHD mouse model.

There was no clear effect of eMSCs-IL10 treatment on body weight (Figure 5B). All the mice in the experiment and control groups had comparable weight regardless of the treatment regimen.

**Figure 6:**
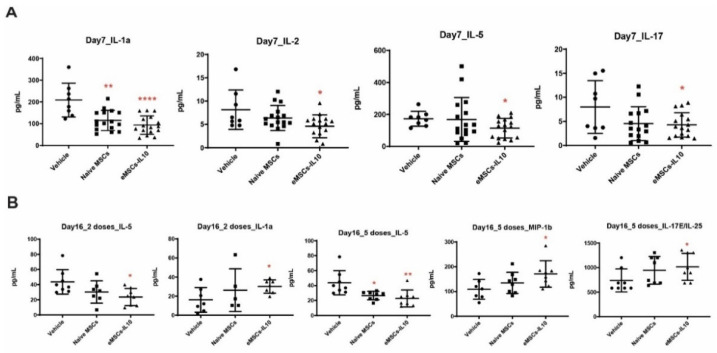
Engineered MSCs-IL10 reduced pro-inflammatory cytokine levels in serum. (**A**) eMSCs-IL10 2-dose treatment regimen significantly reduced serum IL1α, IL-2, IL-5, and IL-17 levels compared to the vehicle group on Day 7. (**B**) Serum IL-5 concentration remained at a low level on Day 16 in the eMSCs-IL10 2-dose regimen group, the eMSCs-IL10 5-dose group, and the naïve MSCs 5-dose group. In contrast, IL-1α level in the eMSC-IL10 2-dose group increased compared to the vehicle group on Day 16, and so did IL-17E and MIP-1β levels in the eMSCs-IL10 5-dose group. Data presented as mean ± SD, * *p* < 0.05, ** *p* < 0.01, and **** *p* < 0.0001.

Compared to the corresponding vehicle control (Figure 5C,D), the eMSCs-IL10 2-dose treatment regimen significantly suppressed the percentages of CD4+ cells and CD8+ cells in the spleen on Day 17 (*p* = 0.03 and *p* = 0.02, respectively). In addition, mice serum cytokines were measured on Day 7 and Day 16. Compared to the vehicle group, the eMSCs-IL10 treatment induced significant reduction of IL-1α, IL-2, IL-5, and IL-17 on Day 7 (*p* < 0.0001, *p* = 0.0156, *p* = 0.0245, and *p* = 0.0317, respectively) (Figure 6A). Notably, IL-5 remained at a significantly low level on Day 16 in the eMSC-IL10 2-dose group, the eMSCs-IL10 5-dose group, and the naïve MSCs 5-dose group (*p* = 0.013, *p* = 0.009, and *p* = 0.013, respectively) (Figure 6B). In contrast, the IL-1α level in the eMSCs-IL10 2-dose group became significantly higher compared to the vehicle group on Day 16 (*p* = 0.027) though the serum IL-1α concentration in all groups was reduced on Day 16 as compared to Day 7. In the eMSCs-IL10 5-dose group, the IL-17E/IL-25 and MIP-1β levels were significantly higher than those of the vehicle group on Day 16 (*p* = 0.047 and *p* = 0.026, respectively). All the other cytokines without significant change in the serum cytokine profiling are presented in Figure S5. Additionally, eMSCs-IL10 infusion did not show any significant change to the proportion of donor CD4+ and CD8+ T cell and pro-inflammatory cytokine levels when compared with infusion of naïve MSCs. Due to the complex cytokine orchestration in the occurrence and treatment of GvHD [31], which may involve different factors at different time points, further investigation is needed to better interpret these results.

Taken together, although naïve MSCs and eMSCs-IL10 failed to rescue the body weight loss, the anti-inflammatory effects of eMSCs-IL10 in the GvHD model were revealed at a cellular level by the reduction of donor CD4+ and CD8+ T cells and suppression of pro-inflammatory cytokine secretion. 

## 4. Discussion

MSC-based therapies have shown great promise in treating different diseases, but extensive variabilities in outcomes have been found in the clinical trials [32]. Therefore, multiple strategies have emerged to enhance the therapeutic efficacy of MSCs, such as genetic modification, preconditioning, and combination therapy [13,33]. In our studies, eMSCs-IL10, which can overexpress IL-10 for at least 4 days and reach their peak secretion on Day 1, showed stably robust expression of functional hIL-10 after cryopreservation, while maintaining MSC inherent properties. Due to the abundant IL-10 secretion from eMSCs-IL10, it may not be necessary for the MSCs to survive in vivo for an extended period.

There has been a debate in the field regarding the impact of cryopreservation on MSC immunomodulatory function [34,35]. Our findings demonstrated that thawed eMSCs-IL10 maintained a high level of IL-10 expression and a high capacity to suppress T cell proliferation. This provides the assurance that eMSCs-IL10 can be banked, thawed, and prepared on the day of administration.

As a well-documented immunosuppressive cytokine, IL-10 exhibits its effects via the induction and maintenance of T cell anergy, the attenuation of T cell proliferation, and the impairment of pro-inflammatory cytokine and chemokine production, such as IL1α, TNF γ, and TNFα [15]. Our in-vitro results indicated that eMSCs-IL10 and CCM-IL10 demonstrated stronger inhibitory effects on T cell proliferation than naïve MSCs and CCM-control, respectively. Furthermore, the immunosuppressive effect of CCM-IL10 was able to be specifically reversed by blocking the IL10 receptor. Although the immunomodulatory functions of MSCs in vitro have been intensively studied, the reported inhibitory effects on T cell proliferation by CCM from naïve MSCs were inconsistent and varied from significant inhibition [36] to limited inhibition [37] to no effect [38]. The contributing factors for these variations could include differences in CCM preparation, dilution factors, and duration of T cell treatment.

From the first report of MSCs being able to produce a striking clinical response in a child with severe aGvHD [39], MSC-based therapeutics have become attractive for both prophylaxis and treatment of aGvHD. While the clinical use of naïve MSCs in aGvHD has been shown to be safe, efficacy is more difficult to establish. Greater consistency in clinical outcomes has been observed in children, whereas the efficacy in adults has been varied [40]. This may be attributed to MSC preparation, MSC dosing, and patient stratification. However, although these factors are very relevant, the potency of the MSCs could be a major contributing factor. Because GvHD pathophysiology is mediated by pro-inflammatory cytokines [41], the potentiation of MSCs through the enhancement of their immunosuppressive capabilities is a promising approach to ameliorate symptoms of GvHD.

There are several prior studies that have evaluated the efficacy of MSC treatment in mouse models of GvHD. Some of them demonstrated improvement of GvHD symptoms following MSC treatment [42,43,44], while others failed to show the benefit of MSC treatment [45,46]. In our study, we observed the inhibition of donor T-cell expansion in the eMSCs-IL10 2-dose group, which suggests that eMSCs-IL10 have enhanced potential to treat GvHD compared to naïve MSCs.

We evaluated two and five eMSCs-IL10 dose treatment regimens and our data revealed that the 2-dose regimen induced more potent immunosuppression than the 5-dose treatment regimen. Additionally, the administrations could limit not only the proliferation of CD4+ and CD8+ T lymphocytes but also the production of some pro-inflammatory molecules (IL1α, IL2, IL5, and IL17). The finding that giving a smaller number of eMSC-IL10 doses was more effective was unexpected, though we and others have observed and reported this phenomenon in stroke and lung transplant rejection applications [47,48]. It is now known that MSCs exert their effects principally through tropism [49]. Since many cytokine effects are concentration-dependent with low and high doses having opposite effects, it is conceivable that giving more MSC doses that exceed the threshold for the desired effect may be limiting or detrimental. More studies will be needed to fully understand this phenomenon.

In addition, eMSC-IL10 treatment significantly reduced the pro-inflammatory cytokines IL-1α, IL-2, IL-5, and IL-17 on Day 7. These cytokines have been shown to be involved in the pathogenesis of GvHD. IL-1α is implicated in the initiation and maintenance of GvHD [50], and neutralizing IL-1α using monoclonal antibodies significantly improved survival in a GvHD mouse model [51]. In contrast, the role of IL-2 in GvHD is very diverse and complicated. In the early phase of GvHD, IL-2 was suggested to have a critical role in the development of GvHD [52,53], while low-dose IL-2 therapy, which stimulates regulatory T cell expansion, has shown benefits in GvHD patients [54]. IL-5, a member of the T helper 2 cytokine family, has been reported to be elevated in the serum of GvHD patients [55]. IL-5, along with IFNγ and TNFα, can be used as predictors of aGvHD severity [56]. In our experiment, the reduction of serum IL-5 level was consistent in eMSC-IL10-treated mice across the dosing groups. Lastly, IL-17 was demonstrated to contribute to the early development of GvHD [57]. Since eMSCs-IL10 suppressed T cell proliferation both in vivo and in vitro, the reduction of these pro-inflammatory cytokines might be a result of the IL10-mediated suppression of T cell activation.

Several studies have suggested that the administration of MSCs engineered to overexpress IL-10 improved the treatment of some diseases in animal models [10,21,22,23,24,25]. MSCs engineered with triple PSGL1/SLeX/IL-10 mRNA were shown to potentiate the MSC-induced anti-inflammatory effects in both a murine inflamed ear model and an EAE model [10,58]. In our study, eMSCs-IL10 exhibited immunosuppressive effects in a mouse model of aGvHD by reducing T cell proliferation and decreasing the production of pro-inflammatory cytokines compared to the vehicle group. However, body weight improvement was not observed, and we did not detect immunomodulatory superiority of eMSCs-IL10 over naïve MSCs in vivo. The limited success of the in-vivo model may be caused by the inadequacies of assessing the therapeutic effects of human MSCs in a murine GvHD model. While CB17/Icr-Prkdc^scid^/CrlCrlj mice lack functional lymphocytes, they do have normal natural killer cells, macrophages, and granulocytes [59], which could attack the donor cells and the injected human MSCs, thereby limiting their lifespan. Additionally, mice and humans have been found to have differing immunology and GvHD pathology [60,61]. In humans, GvHD pathology was found to be globally affected by all immune cells. In contrast, some studies have shown that murine memory T cells were not involved in GvHD induction [62,63]. Another limitation of our study is that MSCs were derived from a single donor. Whether the use of MSCs from different donors leads to variable overexpression of IL-10 needs to be further studied.

## 5. Conclusions

We have demonstrated that MSCs can be engineered to significantly overexpress IL-10 via mRNA transfection while undergoing no changes to their intrinsic phenotype. In addition, we showed that eMSCs-IL10 demonstrate robust immunosuppressive abilities in vitro and in vivo. The 2-dose treatment regimen was superior to the 5-dose treatment regimen in the capacity of eMSCs-IL10 to suppress the expansion of CD4+ and CD8+ T cells and to reduce pro-inflammatory cytokine production in the GvHD mouse model. Although further investigation is needed before using eMSCs-IL10 for human GvHD treatment, it might be a promising approach to treat immune-mediated diseases such as GvHD.

## Data Availability

The data of this study are available from the corresponding author on reasonable request.

## Supplementary Materials

The following are available online at https://www.mdpi.com/article/10.3390/cells10113101/s1. Figure S1: EGFP mRNA can be efficiently overexpressed in MSCs, Figure S2: Representative amplification plots in real-time PCR, Figure S3: Representative flow cytometry analysis for naïve MSCs and eMSCs-IL10, Figure S4: Immunosuppressive properties of CCM-IL10 using cluster formation assay, Figure S5: All the cytokines without significant change in the serum cytokine profiling of GvHD model, Video S1: Representative time-lapse video of PHA-P-only treated groups, Video S2: Representative time-lapse video for PHA-P plus CCM-IL10 treated groups.
