# Supplementary material for: IL-10 mRNA Engineered MSCs Demonstrate Enhanced Anti-Inflammation in an Acute GvHD Model
Source: Cells. 2021 Nov 10;10(11):3101. doi: 10.3390/cells10113101 (PMC8621791; doi:10.3390/cells10113101)
Supplement: Supplementary file 1 [file cells-10-03101-s001.zip › cells-1414795-supplementary.pdf]

Supplementary Figures

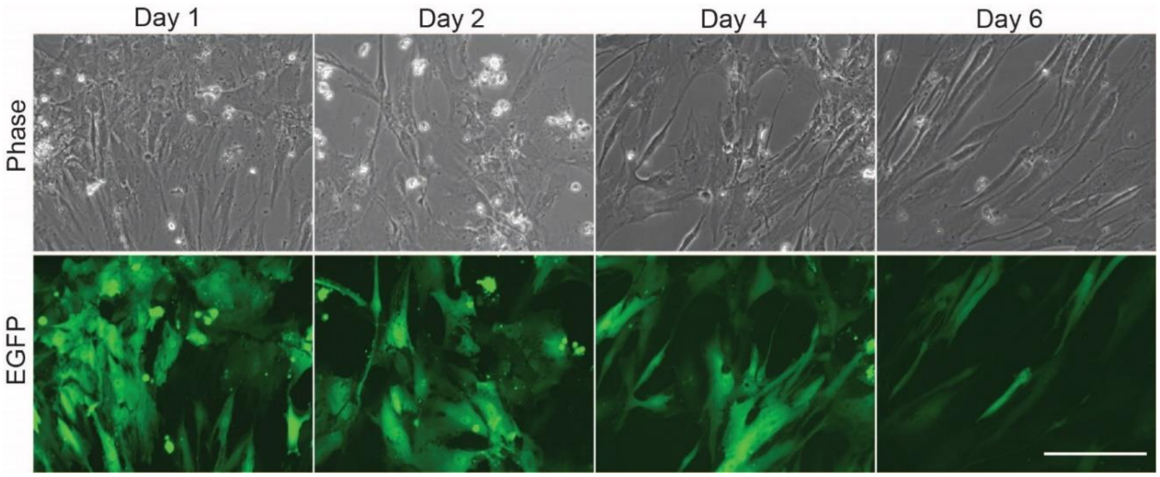

Figure S1 EGFP mRNA can be efficiently overexpressed in MSCs. MSCs were transfected with EGFP mRNA and the images were taken under Phase and GFP channel after 1, 2, 4 and 6 days of transfection. (Scale bar: 200µm).

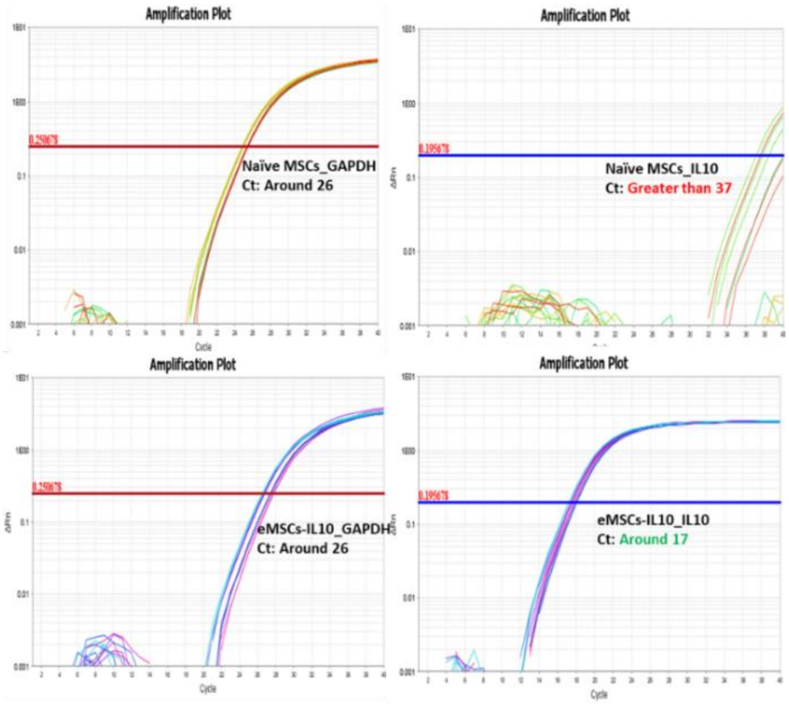

Figure S2 Representative amplification plots in real-time PCR. As an internal control, Ct values of GAPDH remained at roughly 26 in both the naïve MSCs and eMSCs-IL10 groups including samples from the time points of 6 hours, 1, 2, 3, and 4 days. In contrast, the Ct values

of IL-10 were about 17 for eMSCs-IL10 samples whereas IL-10 was barely detectable in the naïve MSCs groups.

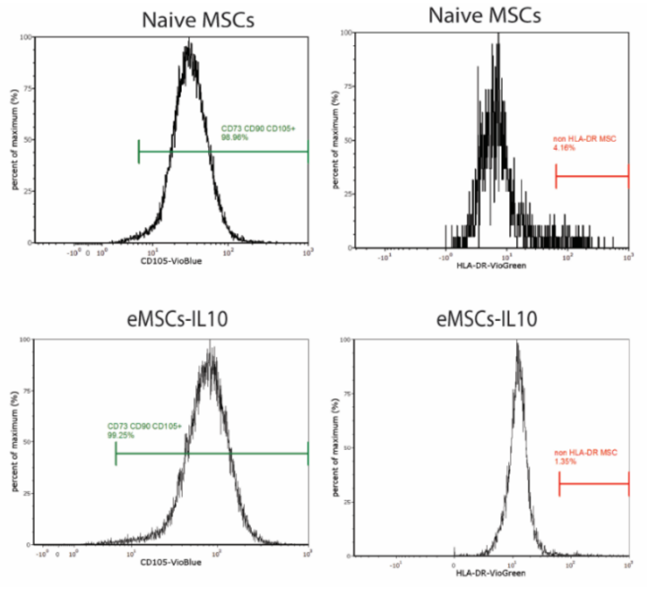

Figure S3 Representative flow cytometry analysis for naïve MSCs and eMSCs-IL10. MSC surface markers were detected by flow cytometry. The analysis of positive markers (CD73, CD90 and CD105) and negative markers (e.g. HLA-DR) was exhibited for naïve MSCs and eMSCs-IL10.

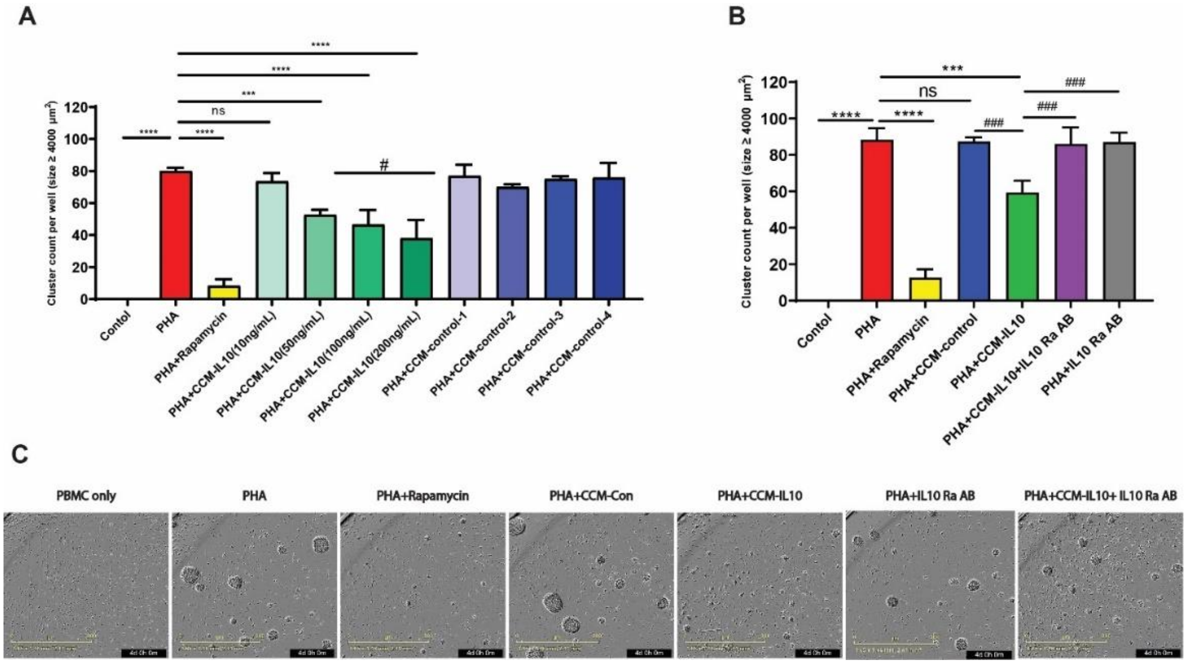

Figure S4 Immunosuppressive properties of CCM-IL10 using cluster formation assay. (A) Different concentrations of IL-10 in CCM-IL10 impact the observed immunosuppressive effects. The immunosuppressive effects were dose-dependent from 10 ng/mL to 200 ng/mL of IL-10,

while respective volume of CCM-control did not cause a significant decrease in cluster number. **(B)** IL-10 receptor α antibody blocked the decrease in cluster number induced by CCM-IL10. **(C)** Representative images of the cluster formation assay. Clusters with an area ⩾ 4000μm2 from PHA, PHA+ CCM-control, PHA+ IL10R α AB and PHA + CCM-IL10 + IL10R α AB group were significantly more numerous than clusters from PBMC only, PHA+ rapamycin and PHA + IL10 group (Scale bar: 800μm). Statistical differences were evaluated by one-way ANOVA. Data presented as mean ± SD (n=3), * indicates groups compared with PHA group and # indicates comparisons with CCM-IL10 treatment groups. *p<0.05, ***p<0.001, ****p<0.0001, #p<0.05, and ###p<0.001.

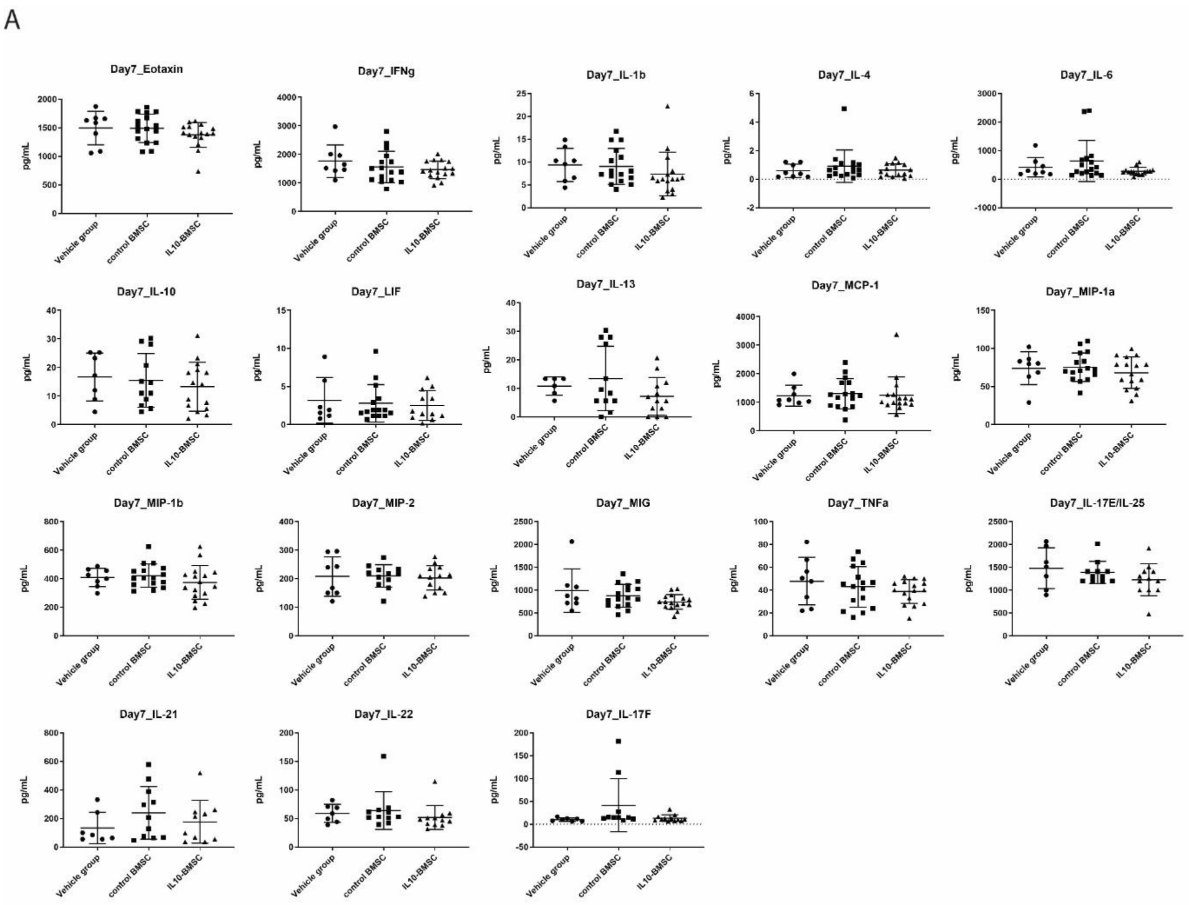

B

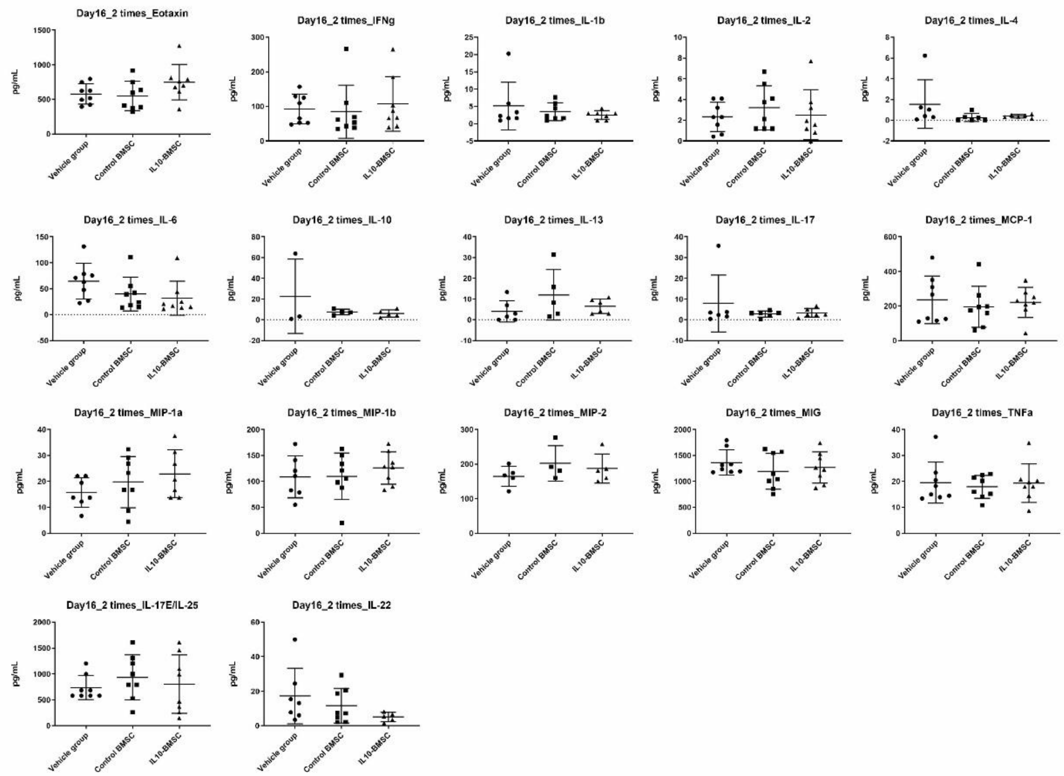
Day16_2 times_Eotaxin
Day16_2 times_IFNg
Day16_2 times_IL-1b
Day16_2 times_IL-2
Day16_2 times_IL-4
Day16_2 times_IL-6
Day16_2 times_IL-10
Day16_2 times_IL-13
Day16_2 times_IL-17
Day16_2 times_MCP-1
Day16_2 times_MIP-1a
Day16_2 times_MIP-1b
Day16_2 times_MIP-2
Day16_2 times_MIG
Day16_2 times_TNFa
Day16_2 times_IL-17E/IL-25
Day16_2 times_IL-22
pg/mL
Vehicle group
Control BMSC
IL10-BMSC

C

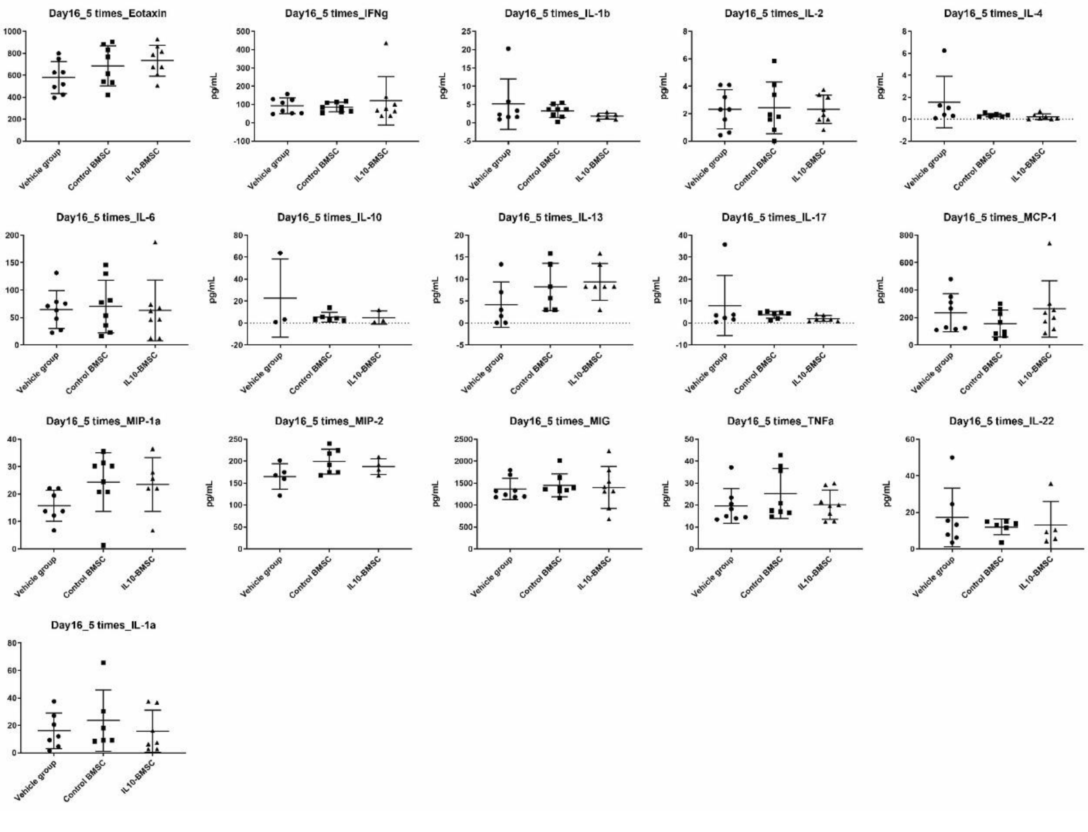
Day16_5 times_Eotaxin
Day16_5 times_IFNg
Day16_5 times_IL-1b
Day16_5 times_IL-2
Day16_5 times_IL-4
Day16_5 times_IL-6
Day16_5 times_IL-10
Day16_5 times_IL-13
Day16_5 times_IL-17
Day16_5 times_MCP-1
Day16_5 times_MIP-1a
Day16_5 times_MIP-2
Day16_5 times_MIG
Day16_5 times_TNFa
Day16_5 times_IL-22
Day16_5 times_IL-1a
pg/mL
Vehicle group
Control BMSC
IL10-BMSC

Figure S5 All the cytokines without significant change in the serum cytokine profiling of GvHD model, (A) on Day 7 (Eotaxin, IFNγ, IL-1β, IL-4, IL-6, IL10, LIF, IL-13, MCP-1, MIP-1α, MIP-1β, MIP-2, MIG, TNFα, IL-17E/IL-25, IL-21, IL-22, IL-17F), (B) on Day 16 in the 2-dose treatment regimen (Eotaxin, IFNγ, IL-1β, IL-2, IL-4, IL-6, IL10, IL-13, IL-17, MCP-1, MIP-1α, MIP-1β, MIP-2, MIG, TNFα, IL-17E/IL-25, IL-22), and (C) on Day 16 in the 5-dose treatment regimen (Eotaxin, I IFNγ, IL-1β, IL-2, IL-4, IL-6, IL10, IL-13, IL-17, MCP-1, MIP-1α, MIP-2, MIG, TNFα, IL-22 and IL-1α).

Video S1 Representative time-lapse video of PHA-P-only treated groups. The images were captured every 4 hours. Scale bar, 800 µm.

Video S2 Representative time-lapse video for PHA-P plus CCM-IL10 treated groups. The images were captured every 4 hours. Scale bar, 800 µm.

Supplementary Method

Cluster formation assay

This assay was conducted using the same protocols and treatments as *2.9. CCM immunosuppressive potency assay* with determining T cell proliferation by cluster number. After seeding PBMCs (Cellero, catalog# 1001, lot# 4498NV19) and adding all the treatments, the 96-well plate was incubated at ambient temperature for 30min, and then placed into the Incucyte S3 (Sartorius) to warm for another 20 minutes prior to scanning. The Incucyte was used to monitor the cell growth using the Phase channel and Whole well scan type for 4 days. After 4 days, the clusters⩾ 4000 µm2 were counted using the Incucyte live-cell analysis system. One-way ANOVA was used for statistical analysis (n=3).
